# Discrimination of Chinese Liquors Based on Electronic Nose and Fuzzy Discriminant Principal Component Analysis

**DOI:** 10.3390/foods8010038

**Published:** 2019-01-21

**Authors:** Xiaohong Wu, Jin Zhu, Bin Wu, Chao Zhao, Jun Sun, Chunxia Dai

**Affiliations:** 1 School of Electrical and Information Engineering, Jiangsu University, Zhenjiang 212013, China; 18352868985@163.com (J.Z.); zhaochao93@163.com (C.Z.); sun2000jun@ujs.edu.cn (J.S.); txdcx@126.com (C.D.); 2 Key Laboratory of Facility Agriculture Measurement and Control Technology and Equipment of Machinery Industry, Jiangsu University, Zhenjiang 212013, China; 3 Department of Information Engineering, ChuZhou Vocational Technology College, Chuzhou 239000, China; wubin2003@163.com; 4 School of Food and Biological Engineering, Jiangsu University, Zhenjiang 212013, China

**Keywords:** electronic nose, Chinese liquors, fuzzy discriminant principal component analysis, K-nearest neighbor classifier, fuzzy set theory, principal component analysis

## Abstract

The detection of liquor quality is an important process in the liquor industry, and the quality of Chinese liquors is partly determined by the aromas of the liquors. The electronic nose (e-nose) refers to an artificial olfactory technology. The e-nose system can quickly detect different types of Chinese liquors according to their aromas. In this study, an e-nose system was designed to identify six types of Chinese liquors, and a novel feature extraction algorithm, called fuzzy discriminant principal component analysis (FDPCA), was developed for feature extraction from e-nose signals by combining discriminant principal component analysis (DPCA) and fuzzy set theory. In addition, principal component analysis (PCA), DPCA, K-nearest neighbor (KNN) classifier, leave-one-out (LOO) strategy and k-fold cross-validation (*k* = 5, 10, 20, 25) were employed in the e-nose system. The maximum classification accuracy of feature extraction for Chinese liquors was 98.378% using FDPCA, showing this algorithm to be extremely effective. The experimental results indicate that an e-nose system coupled with FDPCA is a feasible method for classifying Chinese liquors.

## 1. Introduction

China has a long history of producing and drinking liquors [1]. Chinese liquors are traditionally strong alcoholic beverages, like brandy, vodka, and rum, but their raw materials and production processes are different. Chinese liquors are mainly produced by fermentation of grains (such as sorghum, rice, corn and wheat), followed by distillation and blending [2]. Chinese liquors are the well-liked alcoholic beverages in the Chinese people who like to drink a lot of liquor. Therefore, a large quantity of liquors need to be brewed every year in China. Liquors are also a high value-added commodity, so Chinese liquor factories are of great significance to the Chinese economy [3]. Due to differences in the fermentation process and raw materials, Chinese liquors have their unique aromas and tastes [4]. According to the taste and flavor, Chinese liquors can be classified into several grades. With the development of China’s economy and improvement of the living standard, more and more people are pursuing high-grade liquors, even if their prices are high. In order to gain more profits, some merchants use inferior Chinese liquors to counterfeit high-grade Chinese liquors. The sale of counterfeit Chinese liquors will not only breach the rights and interests of consumers but will also greatly damage the reputation of manufacturers [5]. Inferior Chinese liquors are generally blended directly with alcohol. The content of methanol, aldehydes, and fusel oil in inferior liquors is above the standard allowed by the World Health Organization for drinking. Above all, inferior or fake Chinese liquors are harmful to consumers’ health. Therefore, the classification of Chinese liquors is an important research hotspot, and a rapid detection technology for Chinese liquors has become a critical need in Chinese liquor factories [6,7,8].

At present, traditional methods for detecting liquor quality include sensory analysis and instrumental analysis. Instrumental analysis mainly involves analysis and tests on the substances contained in Chinese liquors through instruments such as mass spectrometry (MS) [9], gas chromatography (GC) [10], GC-MS [11,12], and electronic nose (e-nose) [13,14]. Sensory analysis mainly involves judging the color, aroma, and taste of liquors through the senses of a professional sommelier to determine the quality of the liquor. MS and GC can accurately achieve quantitative analysis, but the cost of the detection process is very high. Although professional sommeliers can also quickly identify different kinds and grades of Chinese liquors, they are susceptible to subjective factors, physical conditions, and the environment, and the results of their analysis are not objective and lack persuasiveness. Compared with MS, GC, and sensory analysis, the e-nose system has many advantages, such as fast detection, easy operation, and low cost.

The e-nose is an analysis and detection technology for food odors [15,16]. It utilizes the cross-sensitivity of sensor array to detect the mixed gas, and it is widely used to analyze the odor of various volatile organic compounds [17,18,19]. The e-nose has been used in many fields, such as food detection [20,21,22], disease diagnosis [23], and environmental monitoring [24,25]. With the continuous development of the e-nose technology, it has also been applied in practice to detect the quality of Chinese liquors. The e-nose can not only quickly classify different grades and kinds of Chinese liquors but also detect inferior or counterfeit Chinese liquors according to their unique flavors.

The e-nose system generally contains four parts: gas collection device, sensor array, signal processing, and machine-learning algorithms. Much research has been done on the application of machine-learning algorithms in the e-nose system for liquors. Principal component analysis (PCA) [26,27] and linear discriminant analysis (LDA) [28] are feature extraction algorithms, and they are applied for reducing the data dimensionality. Recently, researchers combined kernel entropy component analysis (KECA) and LDA with extreme learning machine (ELM) for classification of Chinese liquors by e-nose [29]. Some scholars have proposed a bioinspired breathing sampling algorithm to obtain sample information by e-nose [30]. Random forest (RF) [31,32], LDA, and standard soft-margin C-support vector machines (C-SVM) [33] have been used to identify seven kinds of Chinese liquors by e-nose. Another study combined the RF classifier, quartz crystal microbalance (QCM), and the e-nose system to identify 12 flavors of Chinese liquors [34]. In order to distinguish different geographic origins of Chinese liquors, PCA, hierarchical cluster analysis (HCA), and LDA were employed with e-nose [35]. In addition, the e-nose system has been used for quality testing and flavor evaluation by PCA and cluster analysis [36]. The fuzzy set theory, combined with pattern recognition algorithms, has excellent performance in the electronic nose. The fuzzy c-means (FCM) method was found to be helpful in optimizing the performance and reducing the cost of surface acoustic wave (SAW) electronic noses for detection of milk and fish [37].

In this paper, we designed an e-nose system for the classification of six types of Chinese liquors and developed a feature extraction algorithm, called fuzzy discriminant principal component analysis (FDPCA), by combining discriminant principal component analysis (DPCA) [38] with fuzzy set theory. Compared with DPCA, FDPCA could extract fuzzy features from e-nose signals. In addition to FDPCA, PCA, K-nearest neighbor (KNN) classifier, the leave-one-out (LOO) cross-validation [39], and the k-fold cross-validation were employed in the e-nose system.

## 2. Materials and Methods

### 2.1. Chinese Liquor Samples

We purchased six kinds of Chinese liquors—Maotai (MT), Fenjiu (FJ), Kouzijiao (KZJ), Haizhilan (HZL), Yingjiagongjiu (YJ) and Gujinggongjiu (GJ)—from the local supermarket. The specific information of Chinese liquors is shown in Table 1. We used 50 samples for each type of Chinese liquors, equating to a total of 300 samples for the six types of Chinese liquors. The probability *P*, the difference between samples caused by sampling errors, was less than 0.001 (*N* = 50), indicating the results were statistically significant. There are three flavor types (Maotai style, Fen style, and Luzhou style) in these Chinese liquors. All liquors are produced by solid fermentation, but their origin and raw materials are different. In general, the flavors are different for different type of Chinese liquors. Therefore, we can classify different kinds of Chinese liquors according to their unique flavors using the e-nose system.

### 2.2. Electronic-Nose System

**Figure 1:**
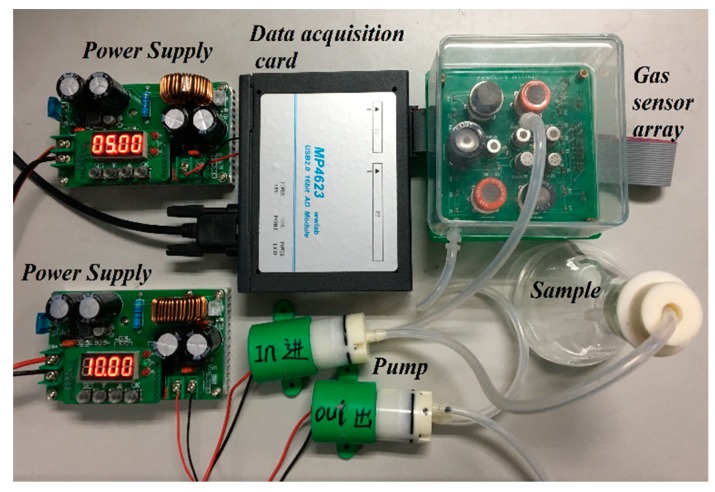
The hardware system of the electronic nose.

The hardware of the designed e-nose system, shown in Figure 1, consisted of a sensor array, power supply, pump (KLP05-6, KAMOER Fluid Technology (Shanghai) Co. Ltd., Shanghai, China), and data acquisition card (MP4623, Beijing Shuangnuo Measurement and Control Technology Co. Ltd., Beijing, China). It had two power supplies, which provided +5 volts and +10 volts for the gas sensor array and the pumps, respectively. One pump was used to transfer the volatile gas of the Chinese liquor samples to the air chamber where the gas sensor array was located. The other pump was used to clean the air chamber. The gas sensor array included 10 kinds of gas sensors: TGS2600, TGS2602, TGS2610, TGS2611, TGS2620, TGS813, TGS822, TGS822TF, MQ136, and MQ3. The Taguchi (TGS) gas sensor, manufactured by Japan Figaro, is a metal-oxide-semiconductor (MOS) gas sensor, which can serve as detectors to measure the current by oxidizing or reducing an object gas at an electrode to obtain a target gas concentration. MQ gas sensors are semiconductor gas sensors manufactured by Weisheng Electronics Technology Co. Ltd. (Zhengzhou, China). The sensitive material of MQ-series gas sensors is a highly active metal-oxide semiconductor.

MQ gas sensors can detect a variety of flammable gases. The MOS sensor is made of a reactive material, such as SnO_2_, ZnO_2_, or Fe_2_O_3_, as the substrate, and a precious metal, such as platinum or palladium, is added as a catalyst. The catalyst can shorten the response time of the sensor to the chemical reaction equilibrium and accelerate the response speed of the sensor. In addition, the MOS sensor has a simple structure and long service life. It is also inexpensive to manufacture and easily miniaturized and integrated. In addition, MOS sensors respond quickly and are reproducible in a short period of time [40]. The details of the sensor parameters are described in Table 2. The gas sensor array could detect the flavors of Chinese liquors and outputted the analog signals to the data acquisition card, which converted the analog signals into the digital signals processed by the computer. The response curves of the digital signals were examined using the LabVIEW 2013 software (National Instruments Corporation, Austin, TX, USA). Machine-learning algorithms for data analysis were programmed with MATLAB 2014 (MathWorks, Natick, MA, USA).

### 2.3. Experimental Steps and Data Processing

**Figure 2:**
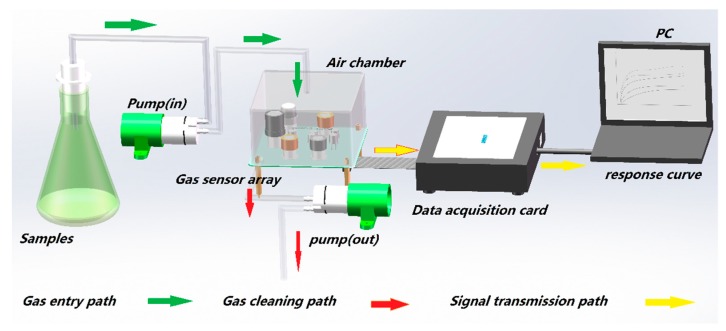
Data acquisition process of Chinese liquors based on electronic nose.

The e-nose data of Chinese liquors were collected in the laboratory (about 15 °C, 40% relative humidity) to ensure the gas sensor worked in an appropriate test environment. The experimental processes are shown in Figure 2.

The experimental steps for data collection of the Chinese liquors using the e-nose system were as follows:

Step 1. Check the electronic nose device, power the e-nose system, preheat the gas sensor array, and open the pump (output) to clean the air chamber.

Step 2. Place 15 ml of Chinese liquor in the conical bottle (where the sample is placed).

Step 3. Send the vapor of the sample to the air chamber by pumping it in (lasting 10 min), collect the data and send them to the PC using the data acquisition card (MP4623), and observe the situation of data acquisition through LabVIEW 2013 software. After 10 min, record and save the data.

**Figure 3:**
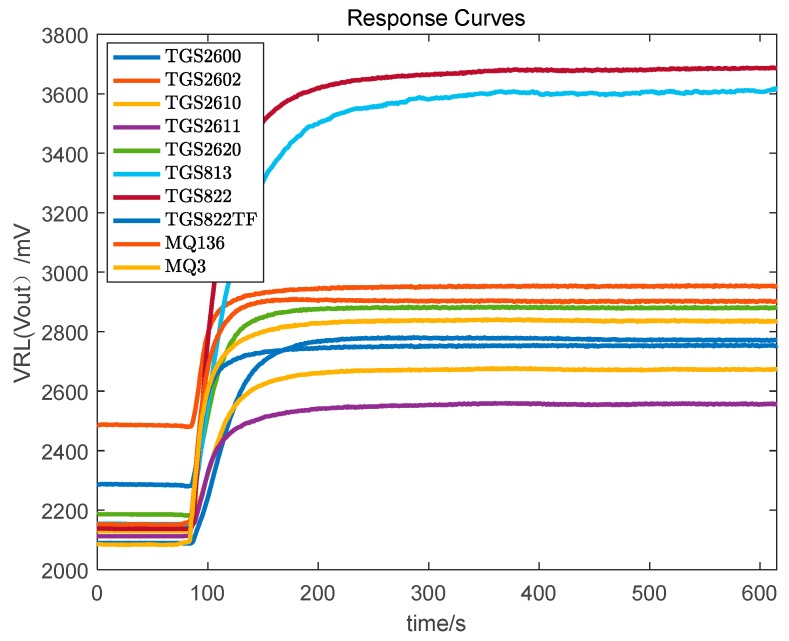
The response curve of pump (input) of Chinese liquors based on sensor array.

**Figure 4:**
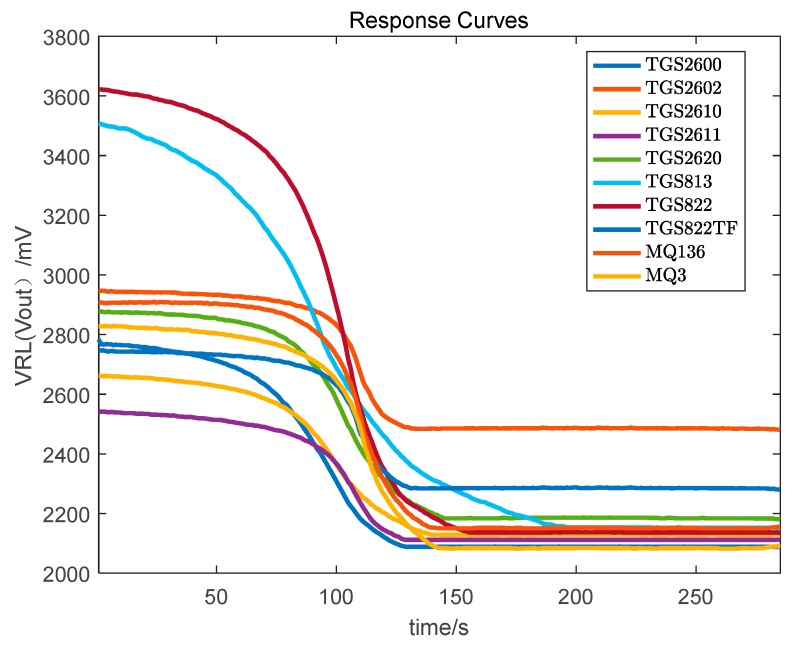
The response curve of pump (output) of Chinese liquor based on sensor array.

Step 4. After the data collection for a sample is complete, clean the gas chamber for 5 min by pumping it out. Whether the gas chamber is clean or not can be observed by the response curve in the LabVIEW 2013 software. After the gas chamber is completely clean, collect the data of another Chinese liquor sample. The response curve of pump (input) and pump (output) of the liquors based on the sensor array is shown in Figure 3 and Figure 4, respectively.

Step 5. Repeat steps 2–4 until all data are collected.

The data processing for Chinese liquor samples included data compression, feature extraction, and data classification. To extract fuzzy features from the data, we used a new feature extraction, i.e. FDPCA, to more accurately classify Chinese liquors. The process of classification using FDPCA was as follows:

Step 1. Compress the data using PCA.

Step 2. Calculate the values of fuzzy membership and the values of cluster centers using fuzzy K-nearest neighbor algorithm.

Step 3. Extract the fuzzy features from the data using FDPCA.

Step 4. Classify the data using the LOO strategy and KNN classifier.

For the LOO strategy, N − 1 samples were selected as training samples from the N samples, and one sample was left to serve as a test sample. Therefore, there were a total of N test samples, and the classification accuracy was calculated N times. In addition, the k-fold cross-validation was used to verify the effect of feature extraction. A total of 30 tests were performed, and the average of the accuracies was calculated.

### 2.4. Discriminant Principal Component Analysis and Fuzzy Discriminant Principal Component Analysis

Based on discriminant principal component analysis (DPCA) [38] and fuzzy set theory, we developed FDPCA to extract fuzzy features. The DPCA algorithm can be described as follows [38]:

Step 1. Calculate the maximum eigenvalue and the eigenvector according to Equation (1):

(1) $$S_{w}^{-1}S_{B}\psi =\lambda \psi$$

where *S_B_* is the between-class scatter matrix; *S_W_* is the within-class scatter matrix; and *Ψ* and λ are the eigenvector and the corresponding eigenvalue, respectively. According to the above calculation, we have the maximum eigenvalue λ_1_ and the corresponding eigenvector *Ψ*_1_, which is the first vector of the optimal discriminant vector set.

Step 2. Calculate a set of optimal discriminant vectors.

(2) $$MS_{w}^{-1}S_{B}\psi_{r+1}=\lambda \psi_{r+1}$$

where

(3) $$M=I-S_{w}^{-1}\psi N^{-1}\psi^{T}$$

$$N=\psi^{T}S_{w}^{-1}\psi$$

$$\psi ={\left[\psi_{1},\;\psi_{2},\ldots ,\psi_{r}\right]}^{T}$$

*I* is the identity matrix; *Ψ_r_*_+1_ and *γ* are the *r* + 1th eigenvector and the corresponding eigenvalue, respectively; and *Ψ*_1_, *Ψ*_2_,…, *Ψ_r_* are a set of optimal discriminant vectors. According to the set of optimal discriminant vectors *Ψ*_1_, *Ψ*_2_,…, *Ψ_r_* (*r* ≥ 1), the next optimal discriminant vector *Ψ_r_*_+1_ can be calculated using Equation (2). The *p* (*p* > *r*) optimal discriminant vectors can be obtained through the above calculation. Then, we have an optimal discriminant vector set {*Ψ*_1_, *Ψ*_2_, …, *Ψ_p_*}.

Before the FDPCA is used for feature extraction, the values of fuzzy membership and the values of cluster centers need to be calculated. The values of fuzzy membership can be obtained through fuzzy K-nearest neighbor algorithms [41]. The values of cluster centers can be achieved using Equation (4):

(4) $$v_{i}=\frac{\sum_{k=1}^{n}u_{ik}^{m_{f}}x_{k}}{\sum_{k=1}^{n}u_{ik}^{m_{f}}},\forall_{i}$$

where $u_{ik}^{mf}$ the fuzzy membership value of $x_{k}$ in the class *i*; $v_{i}$ is the cluster center of the *i*th class; *n* is the number of training samples; ${\bar{x}}_{i}$ is the sample mean of the *i*th class; $m_{f}$ is the weight index, and $m_{f}$ must be greater than one. The feature extraction process of FDPCA is as follows:

Step 1. Calculate the fuzzy between-class scatter matrix $S_{fB}$ and the fuzzy total-class scatter matrix $S_{fT}$ with the following equations:

(5) $$S_{fB}=\overset{c}{\underset{i=1}{\sum }}\overset{n}{\underset{k=1}{\sum }}u_{ik}^{m_{f}}\left(v_{i}-\bar{x}\right){\left(v_{i}-\bar{x}\right)}^{T}$$

(6) $$S_{fT}=\overset{c}{\underset{i=1}{\sum }}\overset{n}{\underset{k=1}{\sum }}u_{ik}^{m_{f}}\left(x_{k}-\bar{x}\right){\left(x_{k}-\bar{x}\right)}^{T}$$

(7) $$\bar{x}=\frac{1}{n}\overset{n}{\underset{j=1}{\sum }}x_{j}$$

where

*c* is the number of class; *n* is the number of training samples;
$\bar{x}$ the mean of training samples;
T represents the transpose of the matrix.

Step 2. Calculate the maximum eigenvalue and the corresponding eigenvector using Equation (8):

(8) $$S_{fT}^{-1}S_{fB}\psi =\lambda \psi$$

where

$S_{fT}^{-1}$ is the inverse of the fuzzy total class scatter matrix;
*Ψ* and *λ* are the eigenvector and the corresponding eigenvalue, respectively. After obtaining the maximum eigenvalue *λ*_1_ and the corresponding eigenvector *Ψ*_1_, suppose that *Ψ*_1_ is the first vector of the fuzzy optimal discriminant vectors.

Step 3. Compute the fuzzy optimal discriminant vectors using Equation (9):

(9) $$PS_{fT}^{-1}S_{fB}\psi_{r+1}=\beta \psi_{r+1}$$

where

$$P=I-\;S_{fT}^{-1}\psi Q^{-1}\psi^{T}$$

$$Q=\;\psi {\;}^{T}S_{fT}^{-1}\psi$$

$$\psi ={\left[\psi_{1},\;\psi_{2},\ldots ,\psi_{r}\right]}^{T}$$

*Ψ_r+_*_1_ is the r+1th eigenvector;
*β* is the corresponding eigenvalue;
*I* is the identity matrix;
*Ψ*_1_, *Ψ*_2_,…, *Ψ_r_* is a set of fuzzy optimal discriminant vectors.

Then, the *r* + 1th fuzzy optimal discriminant vector *Ψ_r_*_+1_ can be obtained according to the fuzzy optimal discriminant vectors *Ψ*_1_, *Ψ*_2_, …, *Ψ_r_* (*r* ≥ 1). Through the above calculation, the *p* (*p* > *r*) fuzzy optimal discriminant vectors {*Ψ*_1_, *Ψ*_2_,…, *Ψ_p_*} can be achieved.

## 3. Results and Discussion

We used FDPCA, DPCA, and PCA to extract features from the e-nose signals of Chinese liquors. Then, K-nearest neighbor (KNN) classifier was employed to classify the data. The LOO cross-validation and k-fold cross-validation were used to verify the performance of the feature extraction methods. Although we used the same classification method, the three feature extraction methods dealt with the e-nose signals separately, and their classification results were different.

### 3.1. Data Preprocessing

When the e-nose is used to acquire data, it is inevitably subject to noise interference, and the collected data cannot be directly used for data analysis. Moreover, when the e-nose is used to collect data for a long time, the collected data may have a certain deviation due to the influence of temperature and humidity (water vapor) on the sensor’s response. Therefore, in order to reduce the impact of these factors on data collection, it is necessary to preprocess the data. First, when the MP4623 was operated for data acquisition, the MP4623_CAL, the data correction function of MP4623, was used for data correction. This could reduce the error during data collection to ensure the authenticity of the data and weaken the influence of temperature and humidity. Second, we took the average value of five different moments in the saturation region of the response curve to remove some noise interference caused by current. Finally, the data were normalized to simplify the complexity of the calculation for the classification of Chinese liquors.

### 3.2. PCA Analysis

**Figure 5:**
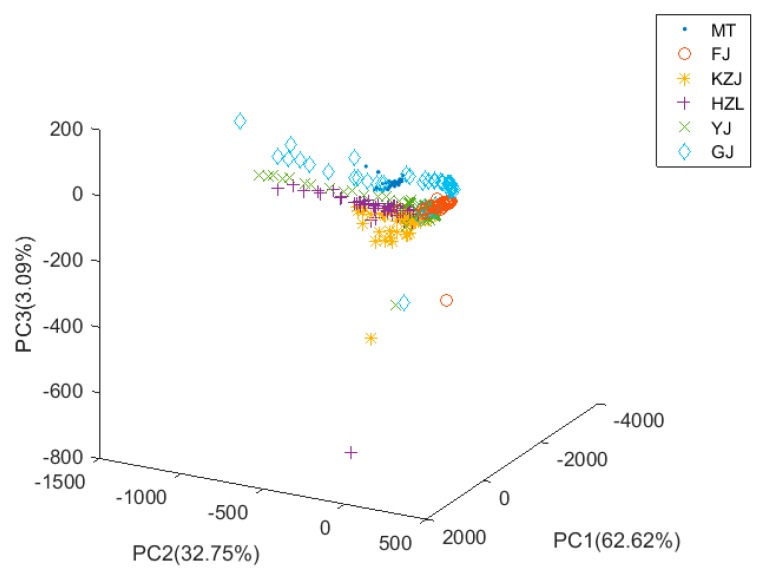
Three-dimensional distribution of data after principal component analysis (PCA). Maotai (MT), Fenjiu (FJ), Kouzijiao (KZJ), Haizhilan (HZL), Yingjiagongjiu (YJ), Gujinggongjiu (GJ).

The PCA is a common statistical algorithm for dimensionality reduction. In this study, the PCA was processed to reduce the complexity of the data and find the most important features. When the PCA was used to extract features, we selected five main features based on the differences in the data. In order to visualize the data information, we drew a three-dimensional scatter plot of the data processed by PCA (Figure 5). The first principal component (PC1) contribution rate was 62.62%, the second principal component (PC2) contribution rate was 32.75%, the third principal component (PC3) contribution rate was 3.09%, and the cumulative contribution rate of the first three principal components was 98.38%. The three-dimensional scatter plot showed that the distances between FJ, HZL, and GJ were relatively far and they could easily be classified. However, the remaining three clusters of Chinese liquors were relatively close and evenly overlapped; as a result, it was difficult to classify them. Finally, we utilized the KNN classifier to classify the data and then used the LOO strategy and k-fold cross-validation to see the performance of the classification. The average cross-validation accuracy of PCA was 89.98%. According to the classification results, the most important features of the e-nose data could be calculated by PCA based on the differences of data (the variance of the data was the largest). However, PCA was not very good at computing the discriminant features for classification, and its classification accuracy was not high.

### 3.3. Classification with DPCA

**Figure 6:**
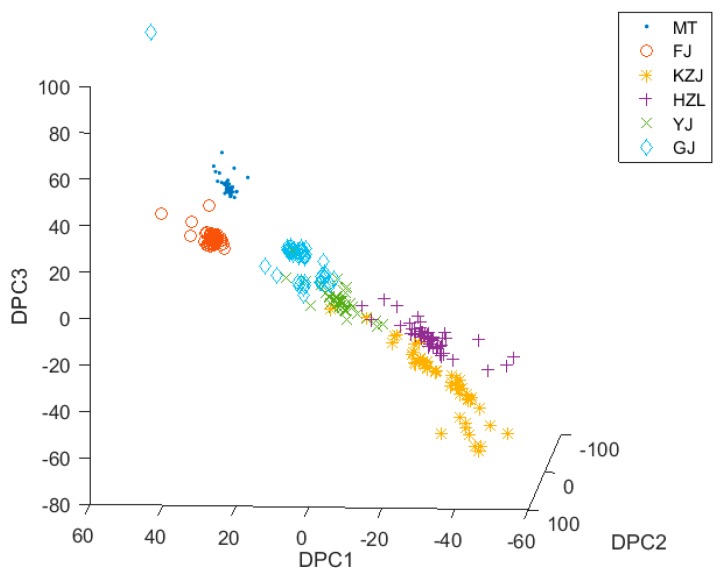
Three-dimensional distribution of data after discriminant principal component analysis (DPCA).

The optimal transformation for discriminant and principal component analysis can be achieved by combining discriminant analysis and principal component analysis. This optimal transformation can reduce the dimensions of data when a set of optimal discriminant vectors is found. Here, we used DPCA for feature extraction to process the e-nose data to obtain an optimal discriminant vector set, which consisted of five optimal discriminant vectors. After the data of Chinese liquors were processed by DPCA, it was easier to classify the data clusters. Figure 6 shows the three-dimensional scatter plot of the data with DPCA. It was found that not only were MT and FJ obviously separated but that HZL and KZJ also had obvious boundaries. The same type of data cluster became more compact, and the distribution of the two different types of data clusters in the three-dimensional space became farther. However, there were also some liquors, such as GJ, HZL, and YJ, whose origins and raw materials were similar. Therefore, these data clusters were very close in three-dimensional space distribution. These data distributions had no obvious boundary lines and even had some overlaps. This would cause great difficulties in classification of GJ, HZL, and YJ. In order to solve this problem, we introduced the fuzzy theory into DPCA, therefore creating the FDPCA. Similarly, we used the KNN classifier to classify the data, and the LOO and the k-fold cross-validation were then used for cross-validation. The average cross-validation accuracy of DPCA was 95.94%, showing it was an appropriate feature extraction method for classification. However, DPCA is a “hard” feature extraction method, not a “soft” one.

### 3.4. Classification with PCA and FDPCA

**Figure 7:**
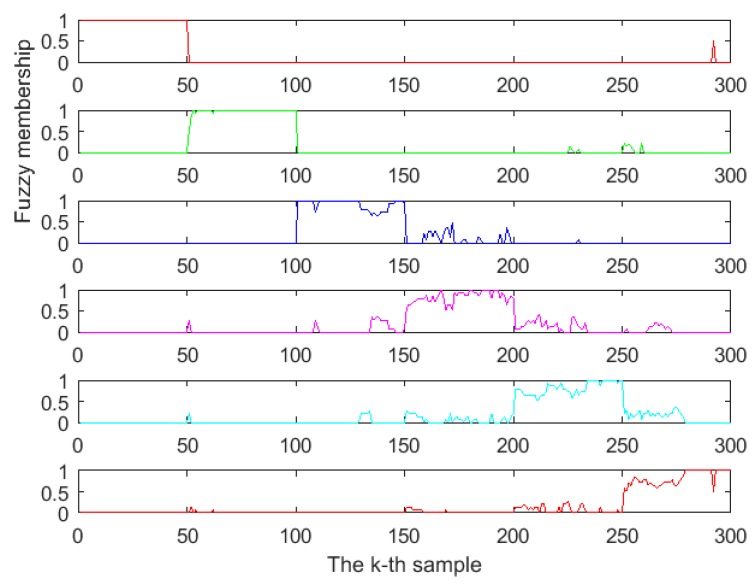
The fuzzy membership values of six Chinese liquor samples.

The FDPCA was developed by introducing fuzzy theory into the optimal transformation that combined discriminant analysis and principal component analysis. When the FDPCA is used for feature extraction, the fuzzy membership ($u_{ik}^{mf}$) and the value of class center ($v_{i}$) should be calculated beforehand. We found that FDPCA worked best when the weight index $m_{f}$ was 2 by comparison. The fuzzy membership values are presented in Figure 7, where the abscissa represents the number of samples (each kind of Chinese liquors had 50 samples), and the ordinate represents the fuzzy membership values. For the kth sample $x_{k}$, if the fuzzy membership value of $x_{k}$, i.e., $u_{ik}$, is more than 0.5, $x_{k}$ belongs to the *i*th class. On the other hand, if $u_{ik}$ is smaller than 0.5, $x_{k}$ does not belong to the ith class. From the six subgraphs in Figure 7, it can be seen that almost all the fuzzy membership values of one kind of liquors were greater than 0.5, while almost all fuzzy membership values of the other kinds of liquors were less than 0.5. This shows that FDPCA based on fuzzy theory was very beneficial for classification. Especially in the areas where the cluster distribution overlapped, FDPCA could assign a weight to those overlapped data points, reduce the complexity of the data structure, and improve the accuracy of classification.

**Figure 8:**
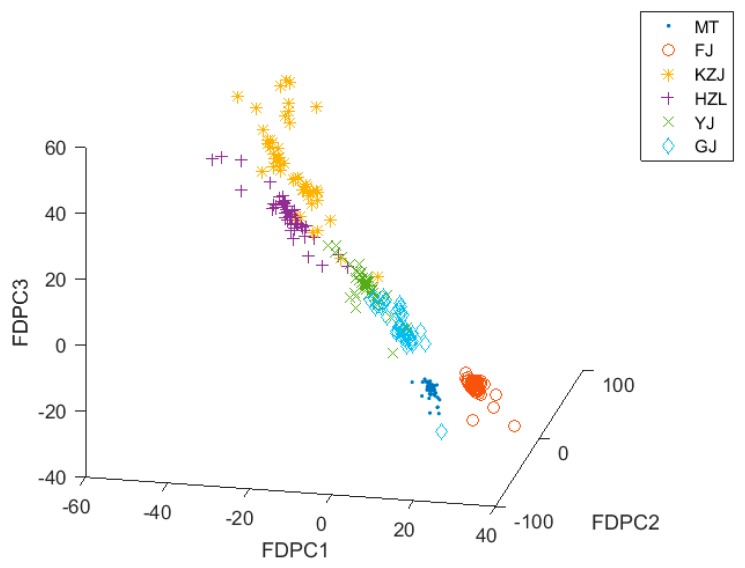
Three-dimensional distribution of data after fuzzy discriminant principal component analysis (FDPCA).

When FDPCA was used for feature extraction, it retained the five important features. Figure 8 shows the distribution of data clusters with FDPCA on a three-dimensional view. As can be seen from Figure 8, the same type of data clusters were more closely aggregated, and the distances between the two different data clusters became farther. The distribution of these data clusters can be largely explained by the differences in the origin and raw materials of different types of Chinese liquors. From the distribution of the data clusters, FDPCA was better than DPCA in feature extraction. Although there were a few data points between HZL, KZJ, and YJ that overlapped, each data cluster was roughly separated. After these data were classified by the KNN classifier with the LOO strategy and the k-fold cross-validation as the cross-validation method, the average cross-validation accuracy of FDPCA was 98.78%.

We used the KNN classifier to classify the data processed by PCA, DPCA, and FDPCA. Then, the LOO cross-validation and k-fold cross-validation (k = 5, 10, 20, 25) were used to verify the accuracy of the classification. The classification results of the three feature extraction methods are shown in Table 3. We found that the average classification accuracy of FDPCA was higher than PCA and DPCA for feature extraction when using the same KNN classifier. According to the accuracy of the validation test, the FDPCA performed well in feature extraction.

As can be seen from Figure 5, Figure 6 and Figure 8, the alcohol content had a significant impact on the classification results. The alcohol content of MT, GJ, and FJ is the same at 53%, and the alcohol content of YJ liquors is 52%, which is almost the same as the alcohol content of the other three kinds of liquors. For liquors with similar alcohol contents, the data clusters were closer or even coincident in the three-dimensional view, making them difficult to be distinguished. However, when the data were processed using the FDPCA algorithm, even MT, GJ, FJ, and YJ were relatively distinguished in three-dimensional view, and classification was relatively easy. This can be seen from the classification accuracy of the verification set in Table 3. After feature extraction with FDPCA, the accuracy of classification was improved by 3.24% on average. This also proved that the advantage of FDPCA was that it could achieve “soft” classification for areas with similar or overlapped data clusters, especially when dealing with a multiclass classification problem. When FDPCA was used for feature extraction, the discriminant information could be effectively obtained, and the classification accuracy was further improved. At the same time, the influence of alcohol content on the classification results was weakened to some extent, thereby improving the ability to identify different types of liquors in the electronic-nose system.

## 4. Conclusions

An e-nose system consisting of 10 kinds of gas sensors was designed to distinguish six different types of Chinese liquors. In addition, in order to improve the performance of feature extraction in distinguishing Chinese liquors, we developed a novel FDPCA algorithm for feature extraction. Through the comparison of classification accuracies, we found that the FDPCA algorithm was better for feature extraction and very useful for improving the performance of the e-nose system in the identification of Chinese liquors. The average classification accuracies of PCA, DPCA, and FDPCA were 89.98%, 95.54%, and 98.78%, respectively, when they were used to extract features from the e-nose data for classification of Chinese liquors. The classification accuracy of FDPCA was obviously higher than PCA and DPCA. Furthermore, we found that FDPCA had advantages in feature extraction when the data structure was complex (where some data clusters overlapped). The classification results show that the e-nose system combined with FDPCA and KNN classifier is a very effective method of identifying different types of Chinese liquors.

## Figures and Tables

**Table 1:** Details of Chinese liquors.

Chinese Liquors	Proof	Raw Material	Place of Origin
Maotai (MT)	53%vol	Sorghum, wheat, water	Zunyi City, Guizhou Province
Gujinggongjiu (GJ)	53%vol	Water, sorghum, rice, wheat, glutinous rice, corn	Haozhou City, Anhui Province
Yingjiagongjiu (YJ)	52%vol	Water, sorghum, rice, corn, wheat	Mianzhu City, Sichuan Province
Haizhilan (HZL)	42%vol	Water, sorghum, rice, corn, wheat, barley, peas	Suqian City, Jiangsu Province
Fenjiu (FJ)	53%vol	Water, sorghum, barley, peas	Fenyang City, Shaanxi Province
Kouzijiao (KZJ)	46%vol	Water, sorghum, corn, rice, wheat, barley, peas	Huaibei City, Anhui Province

**Table 2:** Details of sensor parameters.

Sensor	Target Gas	Standard Test Conditions	
		Circuit Conditions	Preheat Time
TGS2600	Air pollution (hydrogen, alcohol, etc.)	VC = 5.0 +/− 0.01 V DC VH = 5.0 +/− 0.05 V DC	7 days or more
TGS2602	Air pollution (VOC, ammonia, hydrogen sulfide, etc.)	VC = 5.0 +/− 0.01 V DC VH = 5.0 +/− 0.05 V DC	7 days or more
TGS2610	Butane, LP gas	VC = 5.0 +/− 0.01 V DC VH = 5.0 +/− 0.05 V DC	7 days or more
TGS2620	Ethanol, organic solvents	VC = 5.0 +/− 0.01 V DC VH = 5.0 +/− 0.05 V DC	7 days or more
TGS2611	Methane, natural gas	VC = 5.0 +/− 0.01 V DC VH = 5.0 +/− 0.05 V DC	7 days or more
TGS813	Methane, propane, butane	VC = 10.0 +/− 0.1 DC/AC VH = 5.0 +/− 0.05 DC/AC RL = 4.0 kΩ +/− 1%	7 days or more
TGS822	Alcohol, organic solvents	VC = 10.0 +/− 0.1 V DC/AC VH = 5.0 +/− 0.05 V DC/AC RL = 10.0 kΩ +/− 1%	7 days or more
TGS822TF	Coal gas, which includes H_2_ and CO	VC = 10.0 +/− 0.1 V DC/AC VH = 5.0 +/− 0.05 V DC/AC	7 days or more
MQ136	Hydrogen sulfide benzene vapor	VC = 5.0 +/− 0.1 V DC VH = 5.0 +/− 0.05 V DC/AC	More than 48 h
MQ3	Alcohol gas (volatile alcohol)	VC = 5.0 +/− 0.1 V DC/AC VH = 5.0 +/− 0.05 V DC/AC	More than 48 h

LP: liquefied petroleum; VOC: volatile organic compounds; VC: circuit voltage; VH: heater voltage; DC: direct current; AC: alternating current.

**Table 3:** The classification results of the three feature extraction methods.

Types of Models	Feature Number	k (-Nearest Neighbor Algorithm)	LOO Cross- Validation Accuracy	5-Fold Cross-Validation Accuracy	10-Fold Cross-Validation Accuracy	20-Fold Cross-Validation Accuracy	25-Fold Cross-Validation Accuracy	Average Validation Accuracy
PCA	5	7	88.6%	90.44%	90.78%	89.78%	90.28%	89.98%
DPCA	5	7	96%	94.44%	95.56%	95.33%	96.38%	95.54%
FDPCA	5	7	98.33%	98.67%	98.56%	98.89%	99.44%	98.78%

LOO: leave-one-out; PCA: principal component analysis; DPCA: discriminant principal component analysis; FDPCA: fuzzy discriminant principal component analysis.
